# A Role for Auditory Corticothalamic Feedback in the Perception of Complex Sounds

**DOI:** 10.1523/JNEUROSCI.0397-17.2017

**Published:** 2017-06-21

**Authors:** Natsumi Y. Homma, Max F.K. Happel, Fernando R. Nodal, Frank W. Ohl, Andrew J. King, Victoria M. Bajo

**Affiliations:** ^1^Department of Physiology, Anatomy, and Genetics, University of Oxford, Oxford OX1 3PT, United Kingdom, and; ^2^Department of Systems Physiology of Learning, Leibniz Institute for Neurobiology, D-39118 Magdeburg, Germany

**Keywords:** auditory cortex, behavior, chromophore-targeted laser photolysis, ferret, harmonic complex tones, medial geniculate body

## Abstract

Feedback signals from the primary auditory cortex (A1) can shape the receptive field properties of neurons in the ventral division of the medial geniculate body (MGBv). However, the behavioral significance of corticothalamic modulation is unknown. The aim of this study was to elucidate the role of this descending pathway in the perception of complex sounds. We tested the ability of adult female ferrets to detect the presence of a mistuned harmonic in a complex tone using a positive conditioned go/no-go behavioral paradigm before and after the input from layer VI in A1 to MGBv was bilaterally and selectively eliminated using chromophore-targeted laser photolysis. MGBv neurons were identified by their short latencies and sharp tuning curves. They responded robustly to harmonic complex tones and exhibited an increase in firing rate and temporal pattern changes when one frequency component in the complex tone was mistuned. Injections of fluorescent microbeads conjugated with a light-sensitive chromophore were made in MGBv, and, following retrograde transport to the cortical cell bodies, apoptosis was induced by infrared laser illumination of A1. This resulted in a selective loss of ∼60% of layer VI A1-MGBv neurons. After the lesion, mistuning detection was impaired, as indicated by decreased *d*′ values, a shift of the psychometric curves toward higher mistuning values, and increased thresholds, whereas discrimination performance was unaffected when level cues were also available. Our results suggest that A1-MGBv corticothalamic feedback contributes to the detection of harmonicity, one of the most important grouping cues in the perception of complex sounds.

**SIGNIFICANCE STATEMENT** Perception of a complex auditory scene is based on the ability of the brain to group those sound components that belong to the same source and to segregate them from those belonging to different sources. Because two people talking simultaneously may differ in their voice pitch, perceiving the harmonic structure of sounds is very important for auditory scene analysis. Here we demonstrate mistuning sensitivity in the thalamus and that feedback from the primary auditory cortex is required for the normal ability of ferrets to detect a mistuned harmonic within a complex sound. These results provide novel insight into the function of descending sensory pathways in the brain and suggest that this corticothalamic circuit plays an important role in scene analysis.

## Introduction

Investigating how the brain processes complex sounds is essential to understand auditory perception. Along the auditory pathway, there is a clear change in response properties from the inferior colliculus (IC), via the auditory thalamus to the auditory cortex, suggesting that the medial geniculate body (MGB) in the thalamus may be a key stage in transforming or gating the representation of sounds in the brain (Miller et al., 2002; Las et al., 2005; Bartlett and Wang, 2007). Furthermore, its complex serial and parallel connectivity patterns suggest that MGB has a far more complex role in hearing than simply being an obligatory relay station between midbrain and cortex (Sherman and Guillery, 2011; Lee, 2013).

The descending corticothalamic projection from layer VI in primary auditory cortex (A1) to the ventral division of MGB (MGBv) is one of the largest feedback pathways in the auditory system (Rouiller and Welker, 1991; Ojima, 1994; Bajo et al., 1995; Prieto and Winer, 1999), with a key potential role in modulating the receptive field properties and temporal firing patterns of thalamic neurons (He, 1997). Indeed, changes in response properties, such as sharpened tuning curves and shifts in best frequency, have been observed following local inactivation of A1 (Zhang et al., 1997). Cortical stimulation or inactivation experiments have also demonstrated comparable corticothalamic modulation in the visual (Andolina et al., 2007; Wang et al. 2016) and somatosensory (Ghazanfar et al., 2001; Temereanca and Simons, 2004) systems.

Acute pharmacological inactivation of auditory cortex has been demonstrated to yield immediate deficits in the discrimination of basic sound properties (Talwar et al., 2001; Jaramillo and Zador, 2011). However, the extent to which compensatory plasticity occurs following cortical inactivation seems to depend on the spectrotemporal complexity of the stimulus. For example, cortical ablation has little effect on frequency discrimination behavior using pure tones but impairs frequency-modulated tone discrimination (Ohl et al., 1999; Ono et al., 2006; Rybalko et al., 2006; Wetzel et al., 2008). Moreover, previous studies indicate that bottom-up and top-down processing streams are integrated in auditory cortex by recurrent cortico–thalamo–cortical interactions (Barth and MacDonald, 1996; Happel et al., 2014). Hence, disentangling these streams using appropriate manipulation strategies should provide mechanistic insight beyond the information inferred from silencing entire brain regions. In this study, we sought to determine what role corticothalamic feedback might play in auditory perception by examining the effects of selective elimination of corticothalamic projection neurons on the ability of ferrets to detect frequency shifts (“mistuning”) in harmonic complex tones (HCTs).

If two complex tones, such as a pair of vowels, are presented simultaneously, they are more likely to be heard as distinct sounds if their fundamental frequencies differ (de Cheveigné et al., 1997). Harmonicity is therefore an important cue in grouping together the different frequency components of each sound, so that they can be segregated from other concurrent sounds. This can be demonstrated by mistuning one of the frequency components in an HCT, which, in humans, may then be heard as a separate tone with a pitch that differs from that corresponding to the fundamental frequency of the HCT (Moore et al., 1985; Hartmann et al., 1990). The ability to discriminate mistuned complex tones (MCTs) from HCTs has also been measured behaviorally in various nonhuman species (Lohr and Dooling, 1998; Klinge and Klump, 2009, 2010; Homma et al., 2016), and sensitivity to mistuning has been investigated electrophysiologically, both in A1 and at subcortical levels of the auditory pathway (Sinex et al., 2002, 2003, 2005; Sinex, 2008; Fishman and Steinschneider, 2010).

In the present study, the mistuning detection paradigm was combined with chromophore-targeted laser photolysis in pigmented ferrets (*Mustela putorius furo*). This method has been shown to induce apoptosis in retrogradely labeled neurons (Macklis, 1993; Magavi et al., 2000) and can be combined with behavioral measurements (Bajo et al., 2010). By selectively eliminating the unidirectional projection from A1 to the MGBv, we show that corticothalamic feedback contributes to an important aspect of auditory perception.

## Materials and Methods

Twelve adult female ferrets sourced from Marshall BioResources were used in this study (Table 1). All experimental procedures were approved by the local ethical review committee and were performed under license from the UK Home Office in accordance with the Animal (Scientific Procedures) Act (1986, amended in 2012).

**Table 1.** Summary of animals and techniques used in this study

Ferret no.	Recordings in the MGB	Mistuning behavior	Chromophore-targeted laser photolysis
F1418	✓ (Complex tones)		
F1442	✓ (Complex tones)		
F1443	✓ (Complex tones)		
F1205	✓	✓	✓ (A1-MGBv lesion)
F1209	✓	✓	✓ (A1-MGBv lesion)
F1430		✓	✓ (A1-MGBv lesion)
F1223	✓	✓	Control
F1225	✓	✓	Control
F1325	✓	✓	Control
F1433		✓	Control
F1444		✓	Control
F0416			Control (Anatomy)

The check symbol (✓) indicates that the procedures were performed on the animal.

To identify the MGBv, recordings were performed using linear multichannel electrode arrays (NeuroNexus). This information was used to target injections of fluorescent microspheres conjugated with a light-sensitive chromophore into the MGBv to kill descending projection neurons located in layer VI in A1 by laser photolysis (Sheen and Macklis, 1995; Bajo et al., 2010). Animals were tested using a go/no-go behavioral paradigm (Homma et al., 2016) before and after this feedback pathway was removed (Table 1).

### 

#### MGBv identification

##### Surgical procedure.

Anesthesia was induced with a single intramuscular injection of medetomidine hydrochloride (0.022 mg/kg body weight; Domitor, Orion Pharma) and ketamine hydrochloride (5 mg/kg; Narketan10, Vetoquinol), and maintained with an intravenous infusion (3–4 ml/h) of medetomidine hydrochloride (0.022 mg/kg/h) and ketamine hydrochloride (5 mg/kg/h) in 0.9% saline solution supplemented with 5% glucose. Atropine sulfate (0.06 mg/kg, s.c.; Atrocare, Animalcare) and dexamethasone (0.5 mg/kg, s.c.; Dexadreson, Intervet UK) were administered to minimize pulmonary secretions and prevent cerebral edema, respectively. Doxapram hydrochloride (4 mg/kg, s.c.; Dopram-V Injection, Pfizer) was administered to maintain respiratory rate. Perioperative analgesia was provided with buprenorphine hydrochloride (0.01 mg/kg, s.c.; Vetergesic, Sogeval UK) and meloxicam (0.2 mg/kg, s.c.; Metacam, Boehringer Ingelheim). Depth of anesthesia, respiratory rate, EKG, and end-tidal CO_2_ were monitored and maintained throughout the experiment. Temperature was monitored using a rectal probe and was maintained at 38°C using a forced-air warming system (Bair Hugger, 3M Health Care).

The ferret was placed in a stereotaxic frame, the eyes were protected with a carbomer liquid eye gel (Viscotears, Alcon Laboratories), the skull was exposed, and a stainless steel bar was attached above the mid-sagittal ridge using dental cement. After craniotomies were made, animals were moved to an anechoic chamber. Once the dura mater was removed, a single- or double-shank silicon probe (NeuroNexus), with 16 or 16 × 2 recording sites vertically arranged and spaced at 100 μm intervals, was inserted 1.8–2.5 mm anterior to the caudal end of the ectosylvian gyrus and 3.9–4.7 mm lateral from the midline. The probe was slowly advanced perpendicularly to the pial surface while recording neural activity in response to visual and auditory stimuli. The probe was advanced until exclusively auditory responses were encountered (∼9 mm).

##### Stimulus presentation.

Stimulus generation and data acquisition were controlled with a PC using BrainWare software (Tucker-Davis Technologies) and by running customized MATLAB scripts (MathWorks), which communicated with system 3 RP2 and RX5 real-time signal processors (Tucker-Davis Technologies). All auditory stimuli were delivered bilaterally using Panasonic headphone drivers (RPHV297), with an inverse filter generated by closed field calibrations using a one-eighth inch condenser microphone (Type 4138, Brüel and Kjær). Visual stimuli were delivered from an amber light-emitting diode (LED; 2.7 V; light amplitude, 20 mA; diameter, 12.7 mm), located 20 cm in front of the head of the animal.

In the terminal recording experiments (*n* = 3; Table 1), auditory stimuli comprised broadband noise bursts (with a low-pass cutoff frequency of 30 kHz; 100 ms duration; SPL range, 50–90 dB in 10 dB steps), which were sometimes paired with a simultaneous visual stimulus (100 ms), as well as pure tones and complex tones. Pure tones (100 ms, with 5 ms cosine ramps) ranged in frequency from 0.1 to 32 kHz in one-fourth octave steps and between 0 and 80 dB SPL in 10 dB increments. Each frequency-level combination was presented pseudorandomly five or more times at a rate of 1 Hz. To avoid long surgical recovery sessions, a simplified version of the frequency/level combinations (0.5–20 kHz, one-third octave steps, four intensities from 60 to 90 dB SPL) was used in the animals in which chromophore-targeted laser photolysis was performed (Table 1).

Complex tones (350 ms duration) were used in terminal recordings (Table 1). They comprised 16 harmonics with a 400 Hz *F*_0_, referred to hereafter as HCTs. MCTs were generated by shifting the second, fourth, or eighth harmonic to a higher frequency by 0.05%, 0.2%, 0.8%, 3%, or 12%. The complex tones were smoothed by a 25 ms Hanning window at the beginning and end to reduce side lobes in the frequency spectrum. The overall SPLs varied from 40 to 90 dB in 10 dB steps, and each complex tone was presented pseudorandomly 5–10 times at a rate of 1 Hz.

##### Response properties and criteria for MGBv identification.

**Figure 1.**
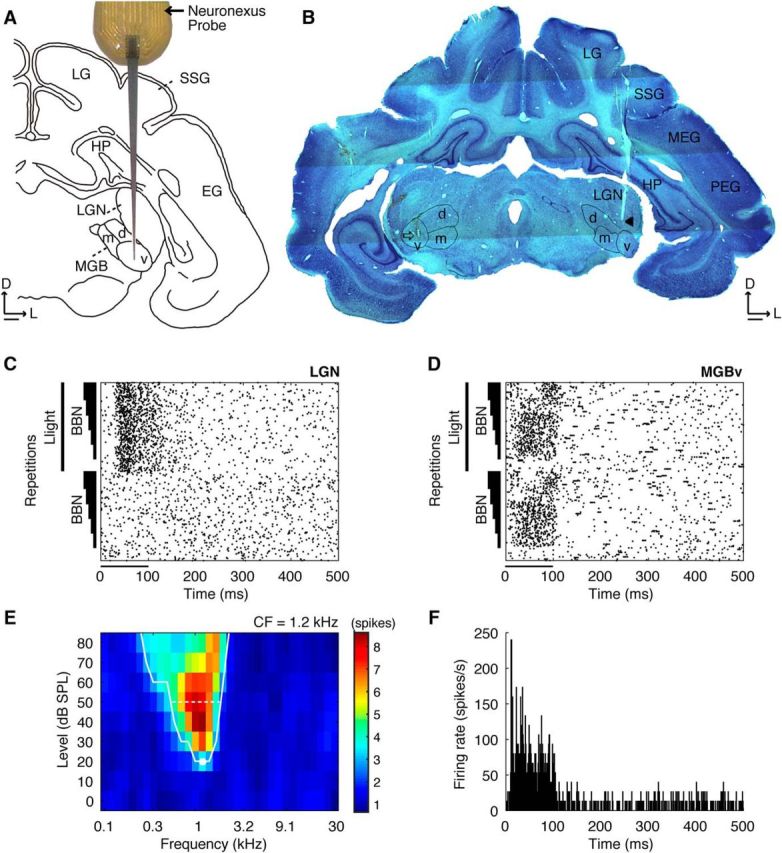
Identification of the ventral division of MGB. ***A***, Schematic of the recording probe (NeuroNexus; 16 recording sites, 100 μm apart) placed dorsoventrally through the cortex into LGN and MGB. ***B***, Coronal section of the midbrain indicating the location of the electrode tracks (arrow in the left MGBv and arrowhead in the right LGN). ***C***, ***D***, Examples of responses to stimulation with broadband noise (BBN) alone or combined with light flashes from an amber LED in the visual (***C***) and auditory (***D***) thalamus. The lines parallel to the *y*-axis indicate the light stimulus, and the triangular bars represent the BBN stimuli increasing in amplitude from 50 to 90 dB SPL in 10 dB steps (20 repetitions). The stimulus duration is indicated by the horizontal bar beneath each plot starting at time 0. ***E***, Example of a sharply tuned V-shaped FRA of multiunit activity recorded in MGBv. The white line indicates the tuning curve. CF is depicted by the white dot, and the white dashed line indicates the bandwidth at 30 dB above threshold. The color scale represents the number of spikes evoked at each frequency-level combination during the 100 ms stimulus. ***F***, Example PSTH of responses to pure tones at unit CF in MGBv. D, Dorsal; d, dorsal MGB; EG, ectosylvian gyrus; HP, hippocampus; L, lateral; LG, lateral gyrus; m, medial MGB; MEG, middle ectosylvian gyrus; PEG, posterior ectosylvian gyrus; SSG, suprasylvian gyrus; v, ventral MGB. Scale bars: ***A***, ***B***, 1 mm.

In a typical penetration (Fig. 1), visual responses in the cortex were first observed up to a depth of ∼4 mm below the pial surface. This activity was followed by large-amplitude spikes with a high spontaneous activity from ∼4 to 5.5 mm deep, which we identified as the hippocampus (Fig. 1*A*,*B*). Strong responses to light flashes from an LED were obtained from ∼5.5 to 7.5 mm below the surface, corresponding to the visual thalamus, the lateral geniculate nucleus (LGN; Fig. 1*C*). Finally, auditory responses to broadband noise were recorded from ∼7.5 to 9 mm deep in the auditory thalamus (Fig. 1*D*). Once acoustically responsive units were identified, a set of pure tones was presented to characterize the frequency tuning of the units. In addition, in three animals (Table 1), we used complex tones to investigate the responses of MGBv neurons to HCTs and MCTs.

Supervised spike sorting and clustering was performed off-line in BrainWare on the basis of different spike features (e.g., amplitude, width and area) and interspike intervals. The spike timing data were exported for further analysis in MATLAB. Only the data where spike counts in a 150 ms window following stimulus presentation for visual stimuli, broadband noise, and pure tones, or a 450 ms window for complex tones, were significantly different from the spike counts in the same duration window at the end of the sweep were analyzed (paired *t* tests, *p* < 0.01). Differences between responses to different stimulus types were confirmed by a one-way ANOVA and *post hoc* Tukey–Kramer tests (*p* < 0.01) for the responses to light flashes and noise bursts. Pooled peristimulus time histograms (PSTHs) were obtained by summing spike counts per bin (1 ms) across stimulus repetitions. Peak response latencies were computed as the time in milliseconds until the highest spike rate was driven in the pooled response, and minimum response latencies were computed as the time until the spike rate first exceeded a criterion defined as 20% of the difference between peak and mean spontaneous firing rates (Bizley et al., 2005).

Frequency response areas (FRAs) were determined from the summed activity for each combination of tone level and frequency (Fig. 1*E*). The threshold at each frequency was used to construct tuning curves using the mean spontaneous rate + 0.2 × (maximum firing rate − mean spontaneous rate) as a response criterion (Sutter and Schreiner, 1991; Bizley et al., 2005; Moshitch et al., 2006), and minimum thresholds and characteristic frequencies (CFs) were determined. Frequency selectivity was estimated by calculating the Q10 (CF divided by the bandwidth at 10 dB above threshold) and BW30 (the bandwidth at 30 dB above threshold).

**Figure 2.**
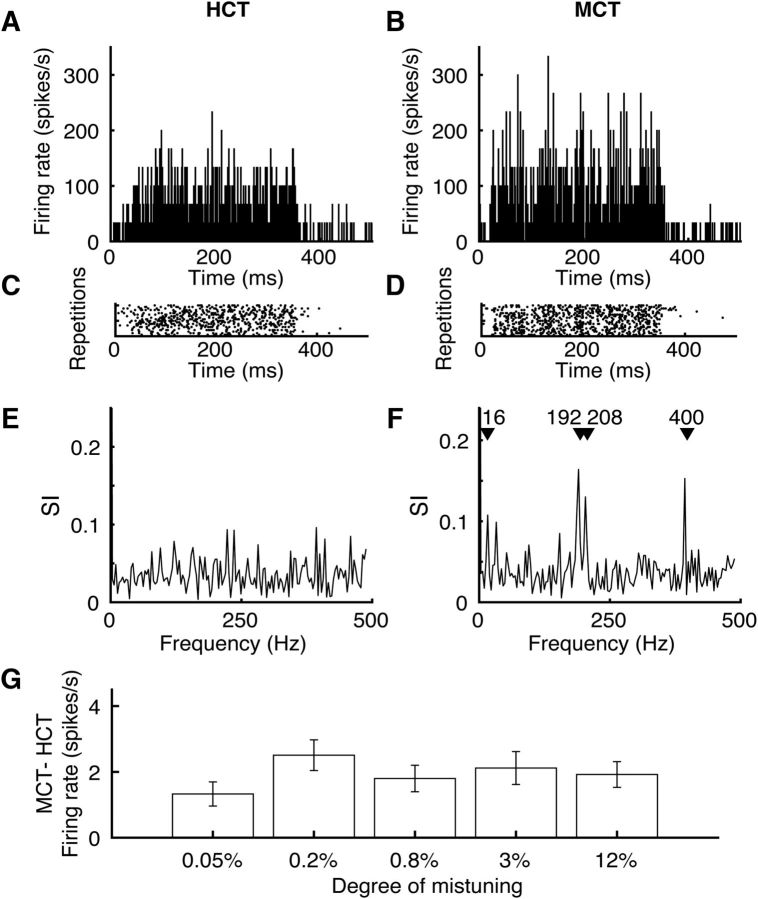
Responses to complex tones in MGBv. ***A***, ***B***, PSTHs showing the responses of one MGBv unit to 350 ms harmonic (***A***) and mistuned (***B***) complex tones. HCTs comprised 16 harmonics with a 400 Hz *F*_0_. In this example, for the MCT, the fourth harmonic was shifted by 192 Hz (12%). ***C***, ***D***, Raster plots corresponding to ***A*** and ***B***, respectively. ***E***, ***F***, Temporal response patterns to HCTs (***C***) and MCTs (***D***) demonstrated using the SI. ***G***, MCTs elicited higher firing rates than HCTs in MGBv units. Bars indicate the difference of firing rate from that evoked by the HCT (mean ± SEM) for each stimulus condition grouped by degree of mistuning (0.05%, 0.2%, 0.8%, 3% or 12%). Firing rate increased when MCTs were presented (repeated-measures ANOVA, *F*_(5,290)_ = 7.4, *p* = 8.2 × 10^−5^), so long as the frequency of the mistuned harmonic fell within the FRA bandwidth of the unit at 30 dB above threshold.

The MGBv limits were established based on the responses to pure tones for units showing sharp tuning curves, clear CFs, and short latencies. In addition, the responses to complex tones in MGBv units were always robust. For units that exhibited a sustained response throughout the duration of the complex tone (73% of the units), the synchronization index (SI; Young and Sachs, 1979) was calculated by performing a fast Fourier transform of the PSTHs for the sustained portion of their response (51–350 ms; Fig. 2).

#### Chromophore-targeted laser photolysis

Chlorin e_6_ monoethylene diamine monoamide disodium salt (Frontier Scientific) was activated with *N-*cyclohexyl-*N*′-(2-morpholinoethyl) carbodiimide methyl-*p*-toluenesulfonate (C106402, Sigma-Aldrich) and attached to the surface of fluorescent microbeads (red and green Retrobeads IX, Lumafluor). The reaction was stopped after 1 h with 0.1 m glycine buffer, pH 8, and a pellet was produced by several periods of high-speed centrifugation (169,537 g) and suspended in PBS.

Injections of active conjugated microbeads were made in the MGBv (red in the left hemisphere and green in the right) in the lesion animals at the stereotaxic coordinates previously determined by recordings. A glass micropipette with a 15–30 μm tip diameter attached to a microinjector (Nanoject II, Drummond Scientific) was placed in a total of five different positions in MGBv, and 50.6 nl was injected by pressure at each position. In control animals, we injected inactive microbeads or just PBS at the same coordinates. In addition, in two other control cases, we injected active conjugated microbeads but in a different location within the brain (for details, see Table 2). One additional control animal (F0416) was used to anatomically assess the relative strength of the projection to the MGBv from different regions of auditory cortex. Ten percent biotinylated dextran amine (BDA; dextran biotin fixable; molecular weight 10,000; Invitrogen) was iontophoretically injected (+5 μA; half duty cycle, 7 s; 15 μm tip diameter; 10 min) at the MGBv coordinates determined from the recording experiments.

**Table 2.** Location and type of injection for the lesion and control groups

Ferret no.	Chromophore-targeted laser photolysis	Injection of microbeads	Injection sites	
			Left	Right
F1205	✓ (A1-MGBv lesion)	✓ (Active)	MGBv	MGBv
F1209	✓ (A1-MGBv lesion)	✓ (Active)	MGBv	MGBd&v
F1430	✓ (A1-MGBv lesion)	✓ (Active)	MGBv	MGBv
F1223	Control	✓ (Inactive)	MGBm	LV
F1225	Control	✓ (Inactive)	LGN	LGN
F1325	Control	PBS	—	—
F1433	Control	✓ (Active)	Hippocampus	PPTg
F1444	Control	✓ (Active)	Rostral pole of IC	Hippocampus
F0416	Control (Anatomy)	BDA	MGBv	—

LV, lateral ventricle; PPTg, pedunculopontine tegmental nucleus. Other abbreviations as in Fig. 1 legend. The check symbol (✓) indicates that the procedures were performed on the animal.

The same surgical procedure was followed as described earlier, but, in addition to the perioperative analgesia provided, amoxicillin and clavulanic acid (20 mg/kg, s.c.; Synulox, Pfizer) were administered over 5 postsurgery days.

A second surgery under isoflurane (1–3% in 1 L of O_2_; IsoFlo, Abbot Laboratories) was performed 3–4 weeks later in lesion and control animals, with the exception of the anatomical control. Craniotomies were made bilaterally over A1 in the middle ectosylvian gyrus (MEG). A1 was exposed to near-infrared light from a laser diode (λ = 670 nm, 280 mW, 10 min; Flatbeam-Laser 670, Schäfter + Kirchhoff) to induce apoptosis in retrogradely labeled cells. The laser light was adjusted with beam-shaping optics (OPTC 5M-G25–60sMA) to create a 1.35 mm spot focused at the level of layer VI, ∼1 mm from the pial surface.

#### Mistuning detection behavior

##### Behavioral paradigm.

**Figure 3.**
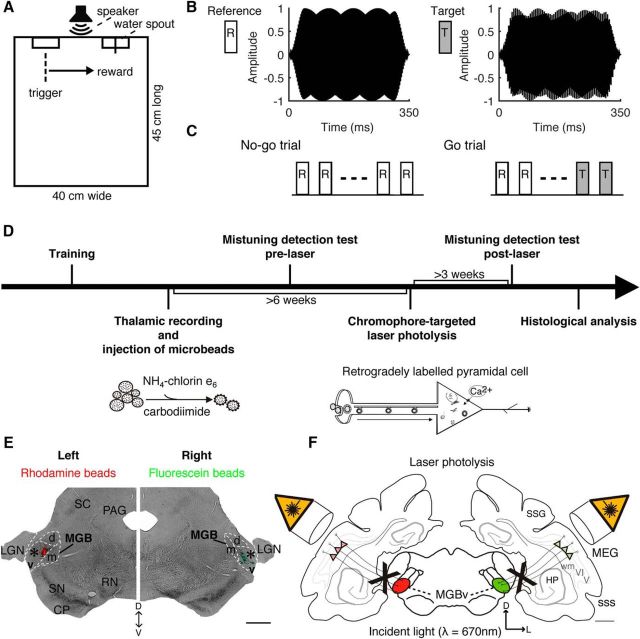
Experimental design. ***A***, The behavioral testing chamber contained two water spouts, labeled as trigger (left) and reward (right) spouts. In between, a loudspeaker was mounted at a height of 20 cm. ***B***, Spectral envelopes of the reference HCT (*F*_0_, 400 Hz; 16 harmonics) and the target tone (MCT, 192 Hz shift in the fourth harmonic). Stimuli were 350 ms long and had a 25 ms Hanning ramp at the onset and offset. ***C***, No-go trials comprised three to seven reference tones, whereas go trials comprised two to six reference tones followed by two target tones. ***D***, After initial behavioral training, the lesion group received injections, guided by electrophysiological recordings, of fluorescent microbeads conjugated with chlorin e_6_ in the MGBv. Sham operations were performed in the controls. After measuring mistuning detection, A1 was exposed bilaterally to near-infrared laser light to induce apoptosis of corticothalamic projection neurons. Three weeks later, mistuning detection was tested again. Finally, brains underwent histological analysis to quantify the corticothalamic lesions. ***E***, Coronal section of one animal at the level of the auditory thalamus, indicating the injection sites of rhodamine (red) and fluorescein (green) microbeads in the left and right MGBv, respectively (asterisks). ***F***, Summary of experimental design for lesioning auditory corticothalamic neurons. V/VI, cortical layers V and VI; PAG, periaqueductal gray; RN, red nucleus; SC, superior colliculus; SN, substantia nigra; sss, suprasylvian sulcus; wm, white matter. Other abbreviations are as in Figure 1 legend. Scale bars, 1 mm.

The animals were trained by positive reinforcement with water reward using a go/no-go paradigm (Homma et al., 2016; Fig. 3). Blocks of 5 or 14 d of training were interspersed with 2 or 3 d off, respectively.

The reference and target stimuli were HCTs and MCTs, respectively, and started in sine phase with a 400 Hz *F*_0_ value and with the same duration and harmonic components previously described (Fig. 3*B*). The target stimulus had the fourth harmonic shifted to a higher frequency. We produced sets of target stimuli with varying degrees of mistuning that initially ranged from 0.5 to 192 Hz in 16 equal logarithmic steps, and later extended this range to include 0.1 Hz over a total of 23 steps.

The animals were first trained to maintain a nose poke at the trigger spout for variable periods of time (0.2–2.0 s) and then to break contact with this spout to obtain a reward at the other spout when two identical target MCTs with the fourth harmonic shifted by 200 Hz were presented (go signal) at 70 dB SPL. In the last part of the training, reference tones (no-go signal) were introduced at an initial level of 30 dB SPL. The level difference between the target and reference tones was gradually reduced as the performance of the animals improved by increasing the level of the reference until all stimuli were presented at 70 dB SPL. The number of reference tones preceding the two target tones on go trials was randomized from two to six to reduce the predictability of the timing of the target tones (Fig. 3*C*; for further details, see Homma et al., 2016).

Trials were self-initiated by the animals by licking the trigger spout. After misses (staying at the trigger spout after the target tones were presented) or early releases (leaving the trigger spout while the reference tones were being presented), a noise burst was played as feedback and to signal the lack of reward. Following a miss, a 1 s time-out was given, whereas a 12 s time-out was used after early releases to reinforce the performance of the animals on trials that required a long waiting time. The probability of trials that comprised reference tones only (no-go trials) was initially 10% and increased to 20% in the last four animals to reduce the number of early releases.

##### Data analysis.

Behavioral performance was assessed by the overall correct response rate (number of correct responses on go trials and correct rejections on no-go trials divided by the total number of trials), hit rate (number of correct responses on go trials/total number of go trials) and false alarm (FA) rate (number of incorrect responses on no-go trials/total number of no-go trials). Individual sessions with FA rates >0.60, likely indicating a lack of attention, were excluded from the analysis.

Using signal detection theory (Wickens, 2002), a sensitivity index (*d*′) was calculated for each degree of mistuning from the *z*-transformed hit rate and FA rate according to the following formula:

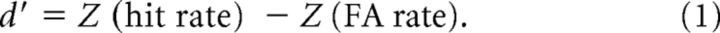
 We estimated psychometric functions by smoothing the *d*′ values based on a model-free estimation (Zychaluk and Foster, 2009), with a weighting function assigned using a triangular kernel function (bandwidth, 40 Hz). The thresholds were defined as the first point to cross the criterion *d*′ = 1, which corresponds to a hit rate of 0.77 with a 0.40 FA rate. Mean psychometric functions were derived by fitting the smoothed *d*′ values using unconstrained nonlinear optimization to a cumulative Gaussian distribution, as follows:

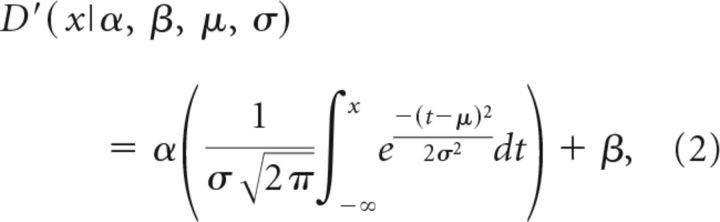
 where *x* is the degree of mistuning (in hertz) on a log scale, α and β are constants to rescale the function in the *y* direction, μ is the mean, and σ the SD.

Response bias was estimated by calculating the following:

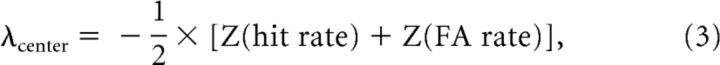
 where λ_center_ is a measure of displacement of the decision criterion. A value of zero indicates no bias in hit and FA rate, whereas positive values imply a lower hit rate and a higher FA rate, and negative values imply a higher hit rate and a lower FA rate.

The area under the curve (AUC) was obtained using the trapezoidal rule for numerical integration over the smoothed *d*′ values on a log scale from the minimum to maximum degree of mistuning used.

#### Histology and stereology

After behavioral testing was complete or at the conclusion of terminal recordings (Table 1), the animals were sedated (0.022 mg/kg, i.m.; Domitor), if awake, overdosed with pentobarbital sodium (2 ml of 200 mg/ml, i.p.; Euthatal, Merial Animal Health) and perfused transcardially with 300 ml of 0.9% saline solution (w/v) and 1 L 4% paraformaldehyde (w/v) in 0.1 m phosphate buffer (PB), pH 7.4.

The brain was dissected from the skull, post-fixed with the same fixative, and cryoprotected with 30% sucrose in 0.1 m PB. Coronal sections, 45 μm thick, were obtained using a freezing microtome (SM2000 R sliding microtome, Leica Biosystems). In animals that only underwent electrophysiological recordings (Table 1), sections were Nissl stained (0.5% cresyl violet) and the positions of the electrode penetrations were confirmed under the microscope. Photographs of the sections were taken using a DMR microscope (Leica Microsystems) equipped with a digital camera (Microfire, Optronics). The anatomical control (F0416) only had one surgery and was perfused 3 weeks after the injection of BDA using the same protocol for the perfusion and preparation of brain sections. To visualize the BDA, sections were incubated with avidin biotin peroxidase (Vectastain Elite ABC kit, Vector Laboratories) and reacted with 0.4 mm 3,3′-diaminobenzidine (DAB; Sigma-Aldrich) enhanced with 2.53 mm nickel ammonium sulfate and 9.14 mm H_2_O_2_ in 0.1 m PB. Finally, sections were Nissl stained.

Serial sections from animals that underwent laser photolysis were divided into five sets. Two sets were processed for fluorescence microscopy, and one set was used to identify neurons by NeuN immunostaining, while the two remaining sets were kept as backups. Sections prepared for fluorescence microscopy were dehydrated in 100% ethanol, cleared with xylene, and coverslipped using a toluene-based mounting medium (HARLECO Krystalon, EMD Chemicals). The location and extent of the microbead injection sites in the thalamus were determined, and the number of retrogradely labeled cells in the auditory cortex was quantified using a fluorescence microscope fitted with filters for fluorescence (448 or 530 nm light emission for green and red, respectively).

NeuN immunostaining was performed on free-floating sections. After permeating the cells with detergent (0.4% Triton X-100 in 0.1 m PBS) and blocking unspecific staining with 5% normal horse serum (Vector Laboratories), sections were sequentially incubated under gentle agitation in primary antibody mouse anti-neuronal nuclei protein (NeuN monoclonal antibody, MAB377, Millipore; dilution, 1:500; 72 h, +5°C), secondary antibody (biotinylated anti-mouse IgG H+L, Vector Laboratories; dilution 1:200; for 2 h at room temperature) and ABC (Vector Laboratories) in 0.1 m PBS. The reaction product was visualized using 0.4 mm DAB and 9.14 mm H_2_O_2_ in 0.1 m PB. The sections were mounted on slides, dehydrated, cleared, and coverslipped with QPath Coverquick 2000 (VWR International).

Unbiased stereological estimation of the number of neurons and volumes of cortical layers were performed using the optical fractionator probe implemented with StereoInvestigator software (version 13, MBF Bioscience). Parameters for stereological estimation were adjusted to obtain a coefficient of error of <0.05 (Gundersen et al., 1988). We performed a nonparametric Mann–Whitney test for the comparison of cell densities across layers, since these were not normally distributed (Shapiro–Wilk test: *W* = 0.95, *p* < 0.05).

## Results

### Responses of MGBv neurons to tones and complex tones

The position of the MGBv was identified based on the response of units to pure tones and the final position of the recording probe in each animal (Fig. 1). Ninety-four units were assigned to MGBv, of which 68 were recorded in the animals used exclusively for electrophysiology (Table 1). Eighty-four per cent of the MGBv units had sharply tuned FRAs (median Q10, 1.3; 25th to 75th percentile range, 0.5–5.8; Fig. 1*E*), a median threshold of 40 dB SPL (25th to 75th percentile range, 40–60 dB SPL), and CFs ranging from 180 Hz to 9.2 kHz. Edeline et al. (1999) found a similar mean threshold of 38 ± 3.1 dB SPL for tones in the MGBv of guinea pigs, while Rodrigues-Dagaeff et al. (1989) reported a threshold range from 30 to 60 dB SPL in cats. Sharper tuning has been observed in guinea pigs (Q10, 2.60 ± 0.25; Edeline et al., 1999) and in squirrel monkeys (0.76 ± 0.85 at <8 kHz and 3.42 ± 2.92 >8 kHz; Allon et al., 1981). However, the Q10 range that we observed largely overlapped that found by Bizley et al. (2005) for neurons recorded in primary areas of ferret auditory cortex. A further 26 MGBv units were recorded in five animals during characterization of the microbead injection sites (recovery surgeries; Table 1) using a reduced number of sound frequency/level combinations (see Materials and Methods).

In both sets of recordings, response latencies to pure tones were short (Fig. 1*F*; minimum latency, median 10 ms; interquartile range, 7–14 ms; median peak latency, 19 ms; interquartile range, 14–25 ms) with no differences between them (Wilcoxon rank sum test: minimum latency, *p* = 0.08; peak latency, *p* = 0.10).

The CFs of units in different recording locations varied systematically with depth in the MGBv, with a tonotopic gradient from high to low frequency along both the anterior–posterior and dorsoventral dimensions of the MGBv. The tonotopicity revealed in these recordings is consistent with the topography of the A1-MGBv projection in ferrets (Nodal et al., 2006) and is similar to that previously described in the cat MGBv (Calford and Webster, 1981; Lee and Winer, 2008).

Harmonic and mistuned complex tones were presented to MGBv neurons using the same stimuli (reference and target tones, respectively) that were used in the mistuning detection behavioral task. A typical example of an MGBv unit (CF, 1.2 kHz) response is shown in Figure 2*A–F*. This unit produced a sustained increase in firing in response to a 400 Hz *F*_0_ HCT (Fig. 2*A*,*C*). When the fourth harmonic was shifted to a higher frequency (by 12%, 192 Hz), the firing rate increased and a distinctive temporal response pattern was observed (Fig. 2*B*,*D*).

Temporal modulation in the responses was analyzed using the SI (Young and Sachs, 1979). The lack of any temporal structure in the PSTH elicited by the HCT (Fig. 2*A*,*C*) was confirmed by a flat plot of the SI versus frequency (Fig. 2*E*). In contrast, when the fourth harmonic of the HCT was shifted, the temporal regularity observed in the PSTH for this unit (Fig. 2*B*,*D*) was associated with SI peaks at 16, 192, 208, and 400 Hz (Fig. 2*F*). The peak at 400 Hz corresponds to the *F*_0_ of the HCT, whereas the other peaks likely reflect interactions between different frequencies in the stimulus. Thus, the peak at 208 Hz corresponds to the frequency of the beat produced by the mistuned and next highest harmonic (2000 − 1792 = 208 Hz). The peak at 192 Hz is the difference in frequency between the *F*_0_ and the first beat (400 − 208 = 192 Hz), and 16 Hz is the envelope periodicity and may result from an interaction between the first and second beat frequencies (208 − 192 = 16 Hz). Similar periodicities have been reported in the chinchilla IC in response to complex tones with a mistuned harmonic (Sinex et al., 2002, 2005).

When the responses of MGBv units to complex tones were analyzed at a population level, the firing rate was found to increase when the frequency of the mistuned harmonic was close to unit CF (Spearman's rank correlation coefficient: *r_s_* = −0.1, *p* = 0.007). We further analyzed the units for which the frequency of the mistuned harmonic fell within their respective FRA bandwidths at 30 dB above threshold. The change in firing rate of these units was significantly different from those units where the frequency of the mistuned harmonic fell outside this bandwidth (two-tailed unpaired *t* test: *p* = 7.3 × 10^−4^). Furthermore, mistuning was identified as a factor in the increase in firing rates (Fig. 2*G*; repeated-measures ANOVA: *F*_(5,290)_ = 7.4, *p* = 8.2 × 10^−5^), with significant differences between the responses to the HCTs and MCTs (Scheffé's test, *p* < 0.05), but not between MCTs with different degrees of mistuning.

These results indicate that neuronal responses in the MGBv are sensitive to mistuning in complex tones, suggesting that the thalamus could be part of the circuitry involved in the detection of harmonicity in complex tones.

### Impairment in mistuning detection after selective elimination of A1-MGBv feedback

We next investigated the role of corticothalamic feedback in mistuning detection. Eight adult ferrets were trained on a mistuning detection task using a go/no-go paradigm (Fig. 3) and subsequently subdivided into two groups, control (*n* = 5) and experimental (*n* = 3). Once experimental animals (hereafter referred to as the corticothalamic lesion group) were trained on this task, bilateral injections of fluorescent microbeads conjugated with chlorin e_6_ were made in the MGBv. Following a survival period of >6 weeks, retrogradely labeled cells in A1 layer VI were ablated by illuminating the auditory cortex on both sides with near-infrared laser light (Fig. 3*D–F*). The control group comprised three different types of controls (*n* = 5; Table 2). Two animals received bilateral injections of active conjugated microbeads in brain areas adjacent to but without encroaching on the MGBv. A further two cases received bilateral injections of inactive microbeads. Finally, one animal received bilateral injections of PBS in MGBv. Subsequent bilateral laser illumination of auditory cortex was performed in all cases as in the experimental group.

**Figure 4.**
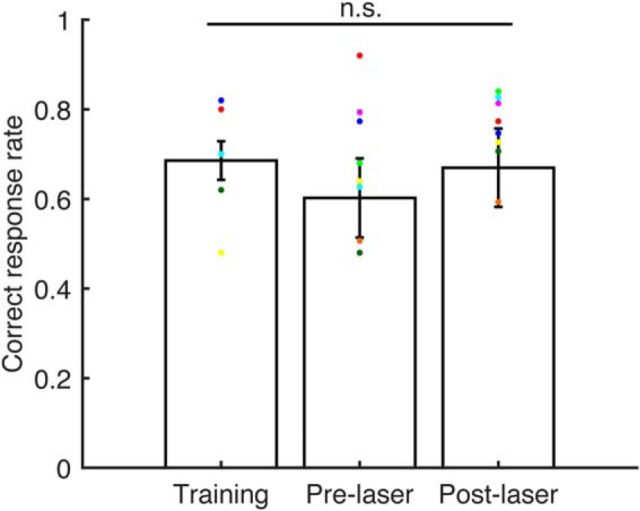
Surgical procedures did not affect the ability of the animals to perform the go/no-go task. Proportion of correct trials when animals had to discriminate MCTs from HCTs with a level difference (<10 dB) between the reference and target tones during the initial training, after injection of microbeads but before laser treatment (Pre-laser), and following laser treatment (Post-laser). Bars represent mean values (±SEM). Colored dots represent individual animals. No difference in performance was found for either the lesion or control group (two-way ANOVA: groups, *F*_(1,17)_ = 1.2, *p* = 0.3; time, *F*_(2,17)_ = 0.5, *p* = 0.6; interaction, *F*_(2,17)_ = 0.9, *p* = 0.4).

To ensure that they were still able to perform the task following the surgical procedures, but without providing further training using near-threshold stimuli, the animals underwent a period of procedural refreshing (∼2 weeks) in which an MCT with the maximum mistuning was used as the target stimulus, reinforced by a level difference between reference and target tones (for details, see training methods). Comparing the performances of the ferrets during these procedural refreshing periods revealed no difference in correct response rates between the lesion and control groups or at different stages of the experiment (training, postmicrobead injections but pre-laser, and post-laser; two-way ANOVA: groups, *F*_(1,17)_ = 1.2, *p* = 0.3; time point, *F*_(2,17)_ = 0.5, *p* = 0.6; interaction, *F*_(2,17)_ = 0.9, *p* = 0.4; Fig. 4).

**Figure 5.**
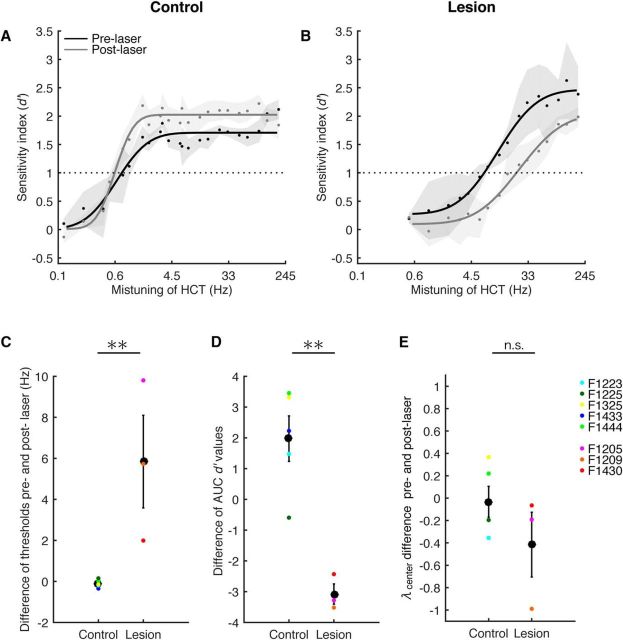
Corticothalamic lesions impaired mistuning detection performance. ***A***, ***B***, Mean *d*′ (±SEM) values derived from the hit and false alarm rates of the control (***A***; *n* = 5) and lesion (***B***; *n* = 3) groups are plotted against the degree of mistuning (on a log scale) of a single-frequency component of an HCT. For each group, data are shown before laser illumination (pre-laser period, black) and >3 weeks after (post-laser period, gray). A cumulative Gaussian distribution was used to fit the psychometric functions (lines). Dots represent mean values across animals, and the shaded areas represent the SEM. The horizontal dashed lines indicate the threshold criterion of *d*′ = 1. ***C***–***E***, Differences (mean ± SEM) between pre-laser and post-laser performance were computed for threshold (***C***), the AUC of the psychometric functions (***D***), and λ*_center_* values (***E***). Colored dots represent data from individual animals. Significant differences between the control and lesion groups are indicated above the panels: **p < 0.01. n.s., Not significant.

Psychometric functions for each animal were obtained, and the *d*′ value was plotted as a function of mistuning (Fig. 5). In control animals, the thresholds did not change between pre-laser and post-laser periods or changes were minimal (±0.2 Hz; bootstrap tests, *p* > 0.05), except for a single case (F1433) in which a lower threshold was obtained post-laser (−0.4 Hz). In addition, small increases in *d*′ values above threshold were observed (Fig. 5*A*). The overall sensitivity to MCTs in controls, indicated by the difference of the AUC of the psychometric functions between pre-laser and post-laser periods, did not change in three of the five cases (Fig. 5*D*; bootstrap tests, *p* > 0.05). When a change was observed (two cases), this was indicative of an increase in sensitivity (Fig. 5*D*). Thus, the mistuning detection ability of the control animals was consistent between pre-laser and post-laser periods, other than a modest improvement in performance in some animals that was presumably training related.

In lesion animals, the thresholds increased (mean ± SEM threshold difference, 5.8 ± 2.3 Hz) and sensitivity decreased (AUC difference, −3.1 ± 0.3) after lesioning of corticothalamic neurons, with the psychometric functions shifted toward higher mistuning values (rightward shift, Fig. 5*B*). In two of the lesion cases (F1205 and F1430), the threshold increased from 7.8 Hz (pre-laser) to 17.8 Hz (post-laser) and from 0.9 Hz (pre-laser) to 2.9 Hz (post-laser), respectively (bootstrap test, *p* < 0.05). In a third case (F1209), the threshold increased from 7.8 Hz (pre-laser) to 13.5 Hz (post-laser), although this difference did not reach significance (bootstrap test, *p* > 0.05). The AUC differences between the pre-laser and post-laser periods decreased significantly for all of the animals in the lesion group (bootstrap tests, *p* < 0.05; Fig. 5*D*), suggesting that their mistuning detection ability had been impaired following the loss of corticothalamic neurons.

These effects were also apparent when we compared the performances of the control and lesion animals. Thus, the threshold change between pre-laser and post-laser periods was significantly larger for the lesion group than the control group (two-tailed unpaired *t* test, *p* = 0.01; Fig. 5*C*). Similarly, the AUC difference in *d*′ values across all mistuning values between pre-laser and post-laser periods differed significantly (mean difference ± SEM: control group, 2.0 ± 0.7; lesion group, −3.1 ± 0.3; two-tailed unpaired *t* test, *p* = 0.002; Fig. 5*D*).

Response bias was estimated by plotting λ_center_ values against the degree of mistuning (0.1–192 Hz) and calculating the slope of the regression line. The slope values tended to be negative for pre-laser and post-laser periods in both groups, indicating a small bias to make “go” responses (Fig. 5*E*). No differences in slope values were found between pre-laser and post-laser periods in either control or lesion groups (slope comparison ANOVA, *p* = 0.1 and 0.9, respectively). Therefore, the mistuning detection impairment exhibited by the corticothalamic lesion group was not due to changes in the decision criteria of the animals.

### Corticothalamic cell loss was observed in animals with impaired mistuning detection

Histological analysis was conducted once animals completed the behavioral testing. The injection sites were identified by the presence of fluorescence (neurons or aggregates of multiple neurons that could not always be individually resolved) that contained the injected microbeads (Fig. 3*E*; Table 2). In lesion cases, the injection sites were mainly restricted to the MGBv, although in one case (F1209, right side) the injection was centered in the dorsal division of the MGB but was spread dorsally in the LGN and ventrally in the MGBv. As expected for MGBv injections, retrogradely labeled fluorescent neurons were always observed in the ipsilateral IC, mainly in its central nucleus. Loss of corticothalamic cells by chromophore-targeted laser photolysis was evaluated using the following two different metrics: by calculating the proportion of fluorescent retrogradely labeled cells in the auditory cortex, and by estimating the neuronal density in each layer of the cortex.

**Figure 6.**
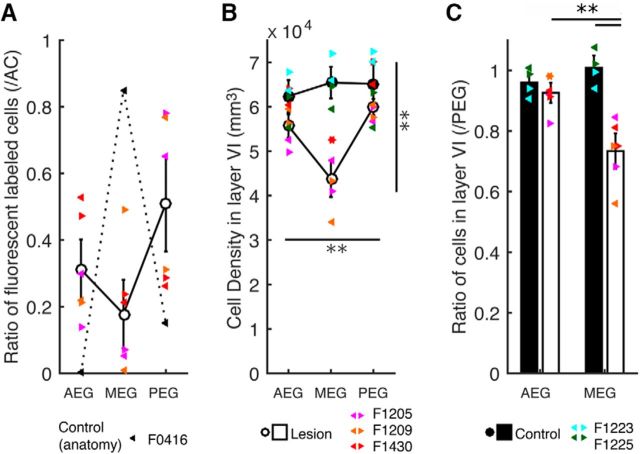
Corticothalamic cell loss after chromophore-targeted laser photolysis in the A1-MGBv pathway. ***A***, The ratio of labeled cells in MEG (where A1 is located), PEG and AEG relative to the total number of labeled cells in the ectosylvian gyrus. White circles indicate the mean in the lesion animals. Black triangles represent values obtained from an anatomical control case in which BDA was injected into the MGBv. Different colors represent individual animals. The left and right pointing triangles indicate data from the left and right hemispheres, respectively. ***B***, Cell density values in layer VI of MEG, PEG, and AEG. Black circles represent the mean in control cases, while white circles indicate the mean in lesion cases. Cell density in the lesion group was significantly different between different auditory cortical regions and from that in the control group (repeated-measures ANOVA: regions, *F*_(2,6)_ = 8.9, *p* = 0.016; region × group, *F*_(2,6)_ = 11.4, *p* = 0.009). ***C***, Histograms of cell density ratio in MEG and AEG (relative to PEG; mean ± SEM) showed significantly lower cell density in the MEG in the lesion group than in the control group (Tukey–Kramer test, **p < 0.01).

The number of retrogradely labeled fluorescent cells in the three main regions of the auditory cortex, the MEG (where A1 is located), posterior ectosylvian gyrus (PEG), and anterior ectosylvian gyrus (AEG), was counted in the lesion animals, and the proportion of cells labeled in each region relative to the total labeled cells in the ectosylvian gyrus was calculated (Fig. 6*A*). These values were then compared with reference values for the ferret A1-MGBv corticothalamic projection obtained from an additional animal used as an anatomical control (F0416), in which a BDA injection was made in the center of the MGBv. In this control case, 85% of the labeled neurons were found in the MEG, 15% in the PEG, and none in the AEG (Fig. 6*A*, dotted line).

All three lesion animals (F1205, F1209, and F1430) had a lower proportion of labeled cells in the MEG than the anatomical control (F0416), suggesting a loss of A1-MGBv projection neurons due to chromophore-targeted laser photolysis (Fig. 6*A*). In addition and contrary to the control case, PEG and AEG, where higher-level auditory cortical fields are located, contained a higher proportion of labeled cells than MEG following five of six MGB injections (Fig. 6*A*). This again suggests that retrogradely labeled neurons were principally lost from A1, the region of auditory cortex that targets MGBv. Only on the right side of F1209 was the proportion of labeling higher in MEG and more like the control, which is consistent with the injection site in this case being located dorsal to the MGBv.

Examination and stereological quantification of NeuN-immunostained sections of the auditory cortex at the level of the MEG revealed a significant reduction in the density in layer VI neurons in the lesion animals relative to the controls (Mann–Whitney test: *U*_(6,4)_ = 24, *p* = 0.005). However, comparison of cell density across other layers did not show any significant differences between lesion and control animals (layer I: *U*_(6,4)_ = 13, *p* = 0.5; layer II/III: *U*_(6,4)_ = 19, *p* = 0.09; layer IV: *U*_(6,4)_ = 19, *p* = 0.09; layer V: *U*_(6,4)_ = 20, *p* = 0.06), confirming that the loss of corticothalamic neurons was restricted to layer VI.

**Figure 7.**
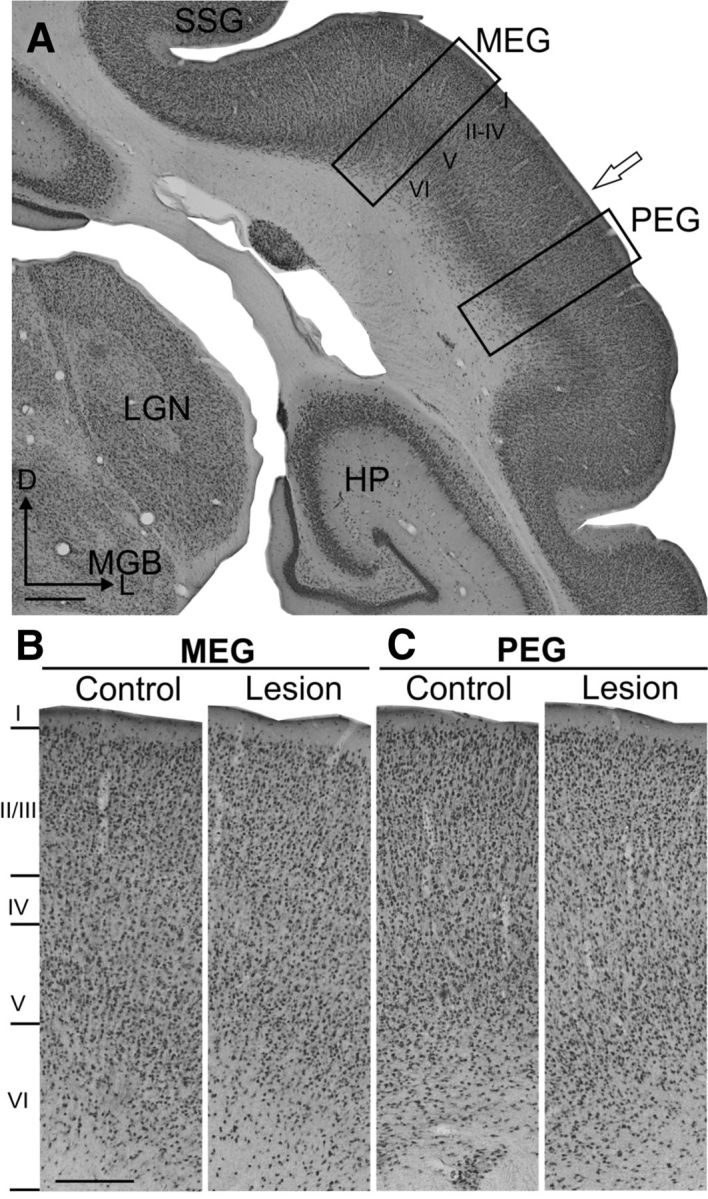
Neuronal density in layer VI of MEG is lower in animals with corticothalamic lesions than in controls. ***A***, Coronal section immunostained with NeuN at the level of the auditory cortex (control case). Rectangles indicate the regions of MEG (where A1 is located) and PEG shown at higher magnification in ***B*** and ***C***. The open arrow indicates the border between MEG and PEG. ***B***, NeuN cell density in MEG layer VI of the lesion example is lower than in the control (for quantification, see Fig. 6). ***C***, No difference in cell density in layer VI of the PEG was found between control and lesion cases. Scale bars: ***A***, 1 mm; ***B***, 250 μm. I–VI, layers 1–6 of the cortex.

We further explored whether there were any changes in layer VI neuron density across groups and cortical regions (Figs. 6*B*,*C*, 7). Stereological quantification of neurons in layer VI across animals demonstrated that the corticothalamic lesion animals had a reduced neuronal density in MEG relative to PEG and AEG, and also to the values seen in control animals (Figs. 6*B*, 7*B*). These differences were statistically significant between lesion and control groups and between cortical regions, but not between left and right hemispheres (repeated-measures ANOVA: cortical regions, *F*_(2,6)_ = 8.9, *p* = 0.016; cortical region × group, *F*_(2,6)_ = 11.4, *p* = 0.009; hemispheres, *F*_(1,3)_ = 8.1, *p* = 0.07, hemispheres × group, *F*_(1,3)_ = 5.3, *p* = 0.1).

No difference in neuronal density in the three regions of auditory cortex were found in the control group, and there were no differences in the values obtained for PEG between lesion and control groups. Therefore, cell densities in AEG and MEG were normalized by the values of PEG to evaluate cell loss (Fig. 6*C*). The proportion for MEG in the lesion group was reduced by 27 ± 4% (mean ± SEM) compared with the control group (Tukey–Kramer test, *p* < 0.01), suggesting that ∼30% of cortical neurons were eliminated by chromophore-targeted laser photolysis.

Collectively, our results show that chromophore-targeted laser photolysis produced selective elimination of layer VI cells in A1 that project to the MGBv, without affecting the number of neurons in the non-primary auditory cortical regions (AEG and PEG).

## Discussion

The present study examined the behavioral consequences of selective lesioning by chromophore-targeted laser photolysis the descending projection from the auditory cortex to the thalamus. We found that the loss of retrogradely labeled corticothalamic layer VI pyramidal neurons that project to the MGBv impaired the ability of ferrets to detect a single mistuned harmonic in an HCT, a measure of sensitivity to the harmonic structure of sounds. Their performance was unaffected, however, on an easier version of the task in which level cues were additionally available for discriminating MCTs from HCTs. These results highlight the importance of the core thalamus (MGBv) in processing complex tones and suggest that corticothalamic feedback contributes to harmonicity detection and, therefore, potentially to auditory scene analysis.

### Responses to complex tones in MGBv

We identified the position of the auditory thalamus by stereotaxic-guided electrophysiology (see also Garcia-Lazaro et al., 2011) and confirmed this by histological reconstruction of recording sites. Response properties of the ferret auditory thalamus were consistent with previous studies in other mammalian species (Allon et al., 1981; Calford, 1983; Rodrigues-Dagaeff et al., 1989; Rouiller et al., 1989; Redies and Brandner, 1991; Bordi and LeDoux, 1994; Edeline et al., 1999; Anderson et al., 2007; Wallace et al., 2007).

MGBv neurons responded to MCTs with specific temporal response patterns. Phase locking corresponding to the periodicities of MCTs has also been reported for neurons recorded in the cochlear nucleus, IC, and A1 (Sinex et al., 2002, 2005; Sinex, 2008; Fishman and Steinschneider, 2010). Furthermore, we found that firing rate in response to MCTs is higher than that in response to HCTs in MGBv neurons when their CFs were close to the frequency of the mistuned harmonic, which is in agreement with findings in primate auditory cortex (Fishman and Steinschneider, 2010).

### Selective elimination of corticothalamic neurons

Chromophore-targeted laser photolysis is a powerful technique for selectively eliminating a targeted population of neurons without damaging other nontargeted neurons, glia, or axons of passage at a particular brain site (Macklis, 1993; Magavi et al., 2000). Following injections of fluorescent microbeads conjugated with chlorin e_6_ into the ferret IC, nonlabeled neurons in other cortical layers are preserved when the infrared laser light is focused at the layer containing the labeled neurons (Bajo et al., 2010). The amount of laser-induced corticothalamic cell loss observed in the present study was comparable to that in previous reports (Eyding et al., 2003; Bajo et al., 2010), with 30% of cells reported to be eliminated in layer VI of the primary visual cortex (V1; Eyding et al., 2003) and 27 ± 4% in the present study.

In cats, corticothalamic cells make up ∼50% of neurons in layer VI of A1 (Prieto and Winer, 1999) and V1 (McCourt et al., 1986; Eyding et al., 2003). Consequently, a 30% loss of neurons in V1 following chromophore-targeted laser photolysis corresponds to ∼60% loss of visual corticothalamic neurons (Eyding et al., 2003). If the proportion of corticothalamic cells in layer VI is the same as in ferret A1, this too would indicate an ∼60% reduction in the number of neurons with descending inputs to the MGBv. This value is close to the previously reported corticocollicular cell loss in ferret A1 using the same approach, which was sufficient to generate a learning deficit in a sound localization task (Bajo et al., 2010).

Although the corticothalamic lesions in our study produced significantly raised mistuning detection thresholds, the animals were still able to perform the task. This may reflect the contribution of intact corticothalamic neurons, as well as other circuits in the auditory system and potential compensatory plasticity.

### Behavioral performance and effect of learning

We measured mistuning detection using a go/no-go design, which has previously been used to show that the threshold for mistuning detecting in ferrets (0.8 ± 0.1 Hz; Homma et al., 2016) is comparable to that reported for other animal species (Lohr and Dooling, 1998; Klinge and Klump, 2009, 2010) and is superior to that measured in humans (Moore et al., 1985; Hartmann et al., 1990; Lohr and Dooling, 1998; Klinge and Klump, 2009). The present study was based on a longitudinal comparison over the course of many months of the performance of individual animals before and after corticothalamic lesion (lesion group) or sham treatment (control group). Importantly, the ability of ferrets to discriminate complex tones on the basis of intensity difference cues was preserved following surgical interventions in both the lesion and control group. Also, control ferrets did not show any impairment in mistuning sensitivity following the sham operations, suggesting that the surgical interventions per se did not affect the behavior of the animals.

The control animals exhibited improved sensitivity for detecting a mistuned harmonic between pre-laser and post-laser periods, although their thresholds remained unchanged. Training-induced changes in A1 response properties have been linked to learning improvements in a number of studies (Kilgard and Merzenich, 1998; Bao et al., 2004; Weinberger, 2004; Ohl and Scheich, 2005; Polley et al., 2006; Schnupp et al., 2006). Hence, it seems likely that neuronal sensitivity to mistuning would have improved in control ferrets over the course of training. In contrast, animals with corticothalamic lesions did not show improved performance over the training procedure but rather a shift of psychometric functions in a direction consistent with impaired mistuning detection and significantly higher thresholds. Therefore, the A1-MGBv feedback pathway appears to be important for the sensitivity of ferrets to the harmonic structure of sounds.

### Corticothalamic feedback and complex sound perception

Corticothalamic feedback from infragranular pyramidal neurons in layers V and VI has a predominantly fast driving or modulatory influence on subcortical neurons, respectively (Ojima, 1994; Zhang and Jones, 2004; Winer et al., 2005). Modulatory layer VI neurons form the majority of the projection from A1 to lemniscal thalamus (Nodal et al., 2006; Llano and Sherman, 2008). Consequently, A1 feedback can influence the properties of MGBv neurons by affecting their overall excitability, receptive field tuning (Zhang and Yan, 2008; Luo et al., 2011), and firing patterns (Ryugo and Weinberger, 1976; Orman and Humphrey, 1981; Villa et al., 1991; He, 1997; He et al., 2002; Yu et al., 2004).

The changes induced by corticofugal feedback in the response properties of neurons in the thalamus and at lower levels of the pathway are thought to be involved in mediating the effects of experience on auditory processing, in turn modifying the information delivered to the cortex (Suga and Ma, 2003; Zhang and Yan, 2008). Although modulation of response properties by corticothalamic feedback has been studied extensively across different sensory modalities (Ghazanfar et al., 2001, Temereanca and Simons, 2004; Andolina et al., 2007; 2013; Wang et al., 2016), the behavioral impact of this top-down circuitry is only now becoming understood (Pais-Vieira et al., 2013; Happel et al., 2014). For example, it has recently been demonstrated that recurrent corticothalamic feedback in rodents promotes the detection of behaviorally important stimuli via a gain modulation of task-relevant information at the level of the auditory cortex (Happel et al., 2014).

Auditory cortical ablations in other species do not impair tone frequency discrimination (Ohl et al., 1999; Ono et al., 2006) but do produce a persistent impairment in the ability of animals to discriminate more spectrotemporally complex auditory stimuli (Rybalko et al., 2006; Wetzel et al., 2008). Consequently, it is unlikely that the raised mistuning detection thresholds produced in the present study by a more selective loss of corticothalamic feedback can be attributed to an effect on tone discrimination. Our results therefore provide the first behavioral evidence that corticothalamic feedback contributes to the perception of complex sounds.

Detection of mistuned harmonics within an HCT may be based on sensitivity to the spatial pattern of excitation or temporal cues (Goldstein, 1973; Houtsma and Smurzynski, 1990; Meddis and Hewitt, 1991a,b), with some evidence suggesting that temporal processing may be more important in nonhuman species (Klinge et al., 2010). In keeping with this, we showed that MGBv neurons in ferrets represent the temporal periodicity of MCTs, suggesting that corticothalamic feedback might be responsible for enhancing temporal precision in the tonic firing patterns of these neurons. While recording from MGBv neurons during a mistuning detection task would be required to confirm these, cortical electrical stimulation or focal inactivation has been shown to shape the temporal response properties of subcortical neurons (for review, see Suga and Ma, 2003). If changes in temporal response properties during behavior are mediated by corticothalamic feedback, this descending projection may also contribute to other tasks that rely on sensitivity to temporal fluctuations, including the segregation of sounds from background noise (Nelken et al., 1999; Las et al., 2005).

It has been particularly difficult to identify the role of corticothalamic modulation in the perception of auditory or other sensory stimuli. By showing that elimination of much of the A1-MGBv pathway degrades the ability of ferrets to perceive the harmonic structure of complex tones, our results suggest that this hitherto poorly understood descending pathway may play a critical role in auditory scene analysis.
